# Abnormal Ultrasonographic Findings of Acellular Dermal Matrix in Implant-Based Breast Reconstruction: Correlations with Histopathology

**DOI:** 10.3390/jcm11041057

**Published:** 2022-02-17

**Authors:** Young Seon Kim, Won Seob Lee, Bo-Yoon Park, Manki Choi, Jun Ho Lee, Young Kyung Bae, Il-Kug Kim

**Affiliations:** 1 Department of Radiology, College of Medicine, Yeungnam University, Daegu 42415, Korea; radkys@yu.ac.kr; 2 Department of Plastic and Reconstructive Surgery, College of Medicine, Yeungnam University, Daegu 42415, Korea; twinreo6542@ynu.ac.kr (W.S.L.); 311plpl@naver.com (B.-Y.P.); man7524@ynu.ac.kr (M.C.); junojunho@gmail.com (J.H.L.); 3 Department of Pathology, College of Medicine, Yeungnam University, Daegu 42415, Korea; ykbae@ynu.ac.kr

**Keywords:** mammaplasty, acellular dermal matrix, ultrasonography, breast implants

## Abstract

Background: Acellular dermal matrix (ADM) in implant-based breast reconstruction can show various ultrasound (US) findings. However, there are limited reports on the US features of the ADM. The aims of this study were to evaluate US findings of the ADM in implant-based breast reconstruction and correlate them with histopathological findings. Methods: Between January 2015 and August 2020, 250 women who underwent implant-based breast reconstruction with ADM and a breast US examination at 6 months to 1 year after reconstruction were retrospectively analyzed. Abnormal US findings were classified as type 1 (focal thickening with decreased echogenicity), 2 (diffusely hyperechoic), or 3 (bright echogenic spots). ADM biopsy was performed in 33 patients who underwent second stage or revisional surgeries. Results: In total, 176 consecutive women with 207 US findings were analyzed. The US findings were normal in 52.2% of the women. The percentages of type 1, 2, and 3 patients were 13.5%, 11.1%, and 23. 2%, respectively. These patients had microscopic findings that showed patchy areas with chronic inflammatory infiltrates, dense collagen bundles without degenerative or inflammatory changes, and empty spaces or degenerated foci unaccompanied by inflammation. Conclusion: Knowing the various ADM presentations on US can help avoid unnecessary invasive procedures.

## 1. Introduction

Breast cancer is the most diagnosed cancer in women worldwide and the leading cause of cancer-related deaths [1]. With early detection and improved treatment, breast cancer-related mortality rates have been decreasing since 1990 [1,2]. As the breast cancer survival rate increases, recovering a patient’s quality of life after cancer is important in breast cancer management.

Breast-conserving surgery with adjuvant radiation therapy is the standard treatment for small breast cancer, but wide resection margins are necessary for oncologic safety, potentially resulting in a deformed breast and asymmetric size compared to the contralateral breast. Mastectomy followed by breast reconstruction can achieve oncological safety, better cosmetic outcomes, and improve patients’ psychological health postoperatively. There are two main techniques for breast reconstruction: using patients’ own tissues, including deep inferior epigastric perforator flap, latissimus dorsi flap, and other autologous flaps, and with implant-based reconstruction. The latter is the most common option following mastectomy [3]. Recently, a major paradigm shift occurred after introducing the acellular dermal matrix (ADM) into breast reconstruction [4,5]. ADM is an extracellular matrix created by the decellularization of cadaveric human tissue, porcine tissue, bovine tissue, or bovine pericardium tissue [6,7,8,9,10]. It can provide structural strength and prevent capsular contracture in implant-based breast reconstruction [11,12]. Breuing et al. first introduced the ADM sling technique to reestablish the lower pole of the pectoralis major muscle that completely encloses the breast implant [13]. ADM use in breast reconstruction provides soft tissue support, implant compartmentalization, and inferior positional control of the pectoralis major muscle during dual-plane reconstruction [5,14]. Recently, prepectoral implant-based breast reconstruction that preserves the pectoralis major muscle has been widely selected and, therefore, the importance of ADM has been highlighted [15,16]. Many previous researchers have demonstrated that reconstructions incorporating ADM improved esthetic outcomes regarding shape, symmetry, and the overall outcome, including contour and position [17,18].

Breast imaging follow-up examination after reconstruction with ADM is usually performed by ultrasound (US). However, there are a few reports on the US features of ADM [6,19,20,21], and only one case report describes the radiologic–histopathologic correlations of ADM used in breast reconstruction [6]. Differentially diagnosing tumor recurrence or fat necrosis from ADM is sometimes challenging because the various ADM presentations on US imaging are not well known.

This study evaluated the ADM US findings and categorized the abnormal findings in implant-based breasts. We also clarified the correlation between abnormal ADM US and histological findings. Knowing the various ADM presentations on US imaging can help avoid unnecessary invasive procedures, such as biopsy or ADM removal.

## 2. Methods

### 2.1. Patients and Clinical Data Collection

The Institutional Review Board of our institute (IRB No. YUMC 2021-03-050) approved this retrospective study and the need for informed consent was waived. All the methods in this study involving human participants were performed following the relevant institutional guidelines and regulations as well as the 1964 Declaration of Helsinki and its lateral amendments or comparable ethical standards. Between January 2015 and August 2020, 250 women who underwent implant-based breast reconstruction with ADM and a breast US between 6 months and 1 year after reconstruction at our institution (a tertiary academic hospital) were included in the review of their medical records. Women were excluded if there was no follow-up breast US examination between 6 months and 1 year after reconstruction (*n* = 34) or if they had no visible ADM on the postoperative breast US (*n* = 40). Finally, 176 consecutive women were included. Data on age, body mass index (BMI, kg/m^2^), type of breast reconstruction, the mean US follow-up period from the reconstruction, the mean implant volume, chemotherapy and radiotherapy history, clinical complications, implant surface type, and ADM manufacturer were collected from the hospital records. Further, 33 of 176 patients underwent two-stage tissue expander-implant breast reconstruction or implant replacement and an ADM biopsy.

### 2.2. Ultrasound Examinations and Analysis

Postoperative breast US imaging was performed by one of three board-certified breast radiologists (HMS, KYS, and LSE with 26, 6, and 3 years of experience in breast imaging, respectively) using a Philips iU22 ultrasound system with a 5–12 MHz linear array transducer or Philips EPIQ Elite ultrasound system with an eL18-4 MHz linear array transducer (Philips Medical Systems, Eindhove, The Netherlands). This study was a retrospective observational study. As our US equipment was changed as of February 2020, the examination before February 2020 was performed with the former US equipment (iU22 with a 5–12 MHz linear array transducer), while the examination after February 2020 was performed with the latter US equipment (EPIQ Elite with eL18-4 MHz linear array transducer). Conventional B-mode ultrasound was used without tissue harmonic imaging or cross beam. One breast radiologist (KYS) retrospectively reviewed the US images of ADM and categorized the findings as normal or abnormal. The abnormal ADM US findings were subcategorized as type 1 (focal thickening with decreased echogenicity), type 2 (diffusely increased echogenicity), or type 3 (bright echogenic spots within the ADM).

### 2.3. Tissue Sample Collection

Thirty-three patients underwent ADM biopsy when they underwent expander-implant exchange (12 patients), implant replacement due to implant rupture (14 patients), or capsular contracture (7 patients). In patients with an abnormal ADM US finding in the breast undergoing second-stage surgery, preoperative skin marking was performed on the area showing abnormal findings on the US. During surgery, 1 × 1 cm^2^ area of the ADM layer below the skin-marked area was excised, and histological examination was performed. A portion of the ADM layer showing normal US findings was also excised (1 × 1 cm^2^ area) after US-guided skin marking for comparison (control). Histological analysis was performed by a pathologist (BYK) using hematoxylin and eosin staining. The US and histologic findings were directly compared.

### 2.4. Statistical Analyses

Statistical analyses were performed using the SPSS statistical software version 22 (IBM Corp., Armonk, NY, USA). To identify independent predictors of risk for abnormal ADM findings, multiple logistic regression models were tested to control for the confounding effects of each variable. In the analysis, type 1, 2, and 3 patients were grouped according to their “abnormal US ADM findings”. Moreover, 95% confidence intervals (CIs) were derived, and *p* values < 0.05 were considered statistically significant.

## 3. Results

### 3.1. Abnormal US Findings Classification

**Figure 1:**
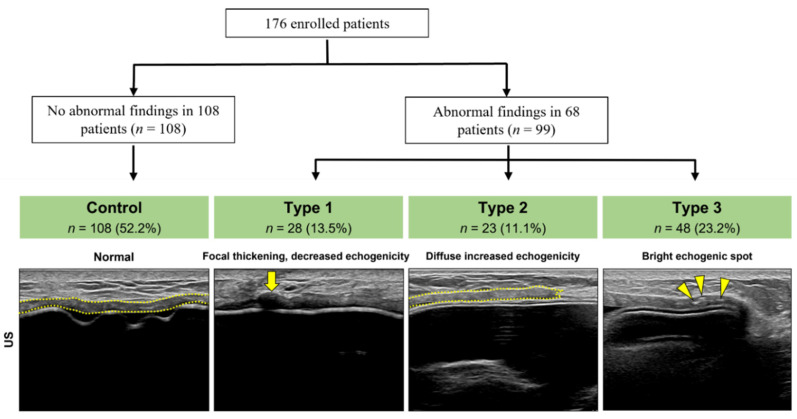
Categorizing ultrasound (US) finding of the acellular dermal matrix (ADM, yellow dotted line) in implant-based breast reconstruction (176 patients, 207 US findings). Normal ADM (control) shows consistent thickness with homogeneous echogenicity. Abnormal findings were classified as: type 1: ADM shows focal thickening with decreased echogenicity (arrow); type 2: diffusely increased echogenicity; and type 3: bright echogenic spots (arrowheads) within the ADM.

In total, 176 patients with 207 US findings were analyzed; 52.2% (*n* = 108) were normal. The ADM on US showed consistent thickness with homogeneous echogenicity. Overall, 13.5% (*n* = 28) of the abnormal findings were classified as type 1, 11.1% (*n* = 23) were classified as type 2, and 23.2% (*n* = 48) were classified as type 3 (Figure 1).

### 3.2. Histologic Findings by Type

**Figure 2:**
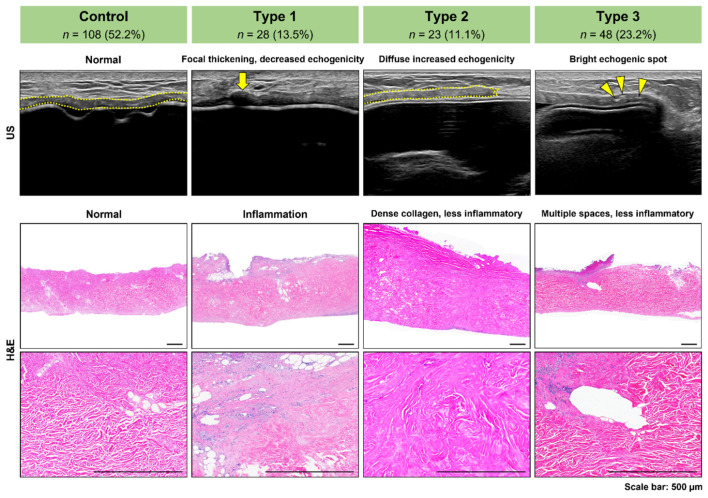
Correlations between the acellular dermal matrix (ADM) ultrasound and histopathologic findings. Histologically, type 1 shows patchy areas with chronic inflammatory infiltrate with a few foreign-body giant cells and neovascularization. Type 2 shows dense collagen bundles without degenerative or inflammatory changes and a few cells. Type 3 shows multiple empty spaces inside the ADM.

We evaluated the correlations between the US and the histologic findings based on the abnormal-type classification. For type 1, microscopic findings showed patchy areas with chronic inflammatory infiltrate with a few foreign-body-type giant cells and neovascularization (Figure 2). Fat cells and fat necrosis in the ADM and surrounding histiocytes were also observed, which were considered to result from a reaction caused by small fat tissues in the ADM itself. Dense collagen bundles without degenerative or inflammatory changes were observed in type 2 (Figure 2) with few cells. Empty spaces or degenerated foci unaccompanied by inflammation were observed in type 3 cases (Figure 2). Calcification was never observed.

### 3.3. Patient Characteristics and Clinical Complications

Among 176 patients, 164 underwent direct-to-implant (DTI) breast reconstruction and 12 patients underwent two-stage expander breast reconstruction. Among the DTI breast reconstruction cases, 21 and 143 were prepectoral, and dual-plane subpectoral breast reconstructions, respectively. All tissue expanders were placed in the subperal plane. Table 1 summarizes the results of the analysis of the clinical characteristics by dividing the patient groups according to the US findings. BMI, radiotherapy, implant volume, and complications did not differ between groups. Age (odds ratio (OR), 1.074; 95% CI, 1.022–1.129), chemotherapy (reference: no chemotherapy; OR, 2.792; 95% CI, 1.272–6.127), microtextured (reference: macrotextured; OR, 5.403; 95% CI, 1.284–22.736), and CGCryoderm (DaeWoong Bio Incorporated, Seoul, Korea) (reference: Megaderm (L&C BIO Inc, Seoul, Korea); OR, 8.167; 95% CI, 3.722–17.922) were significant predictors of abnormal ADM US findings (Table 1).

The mean age of the participants in the control group was 46 years, and the mean type 1, 2, and 3 group ages were 49, 52.1, and 49.4 years, respectively. The implant surface was macrotextured in 84.3% of those in the control group, and in 53.6%, 78.3%, and 62.5% of those in type 1, 2, and 3 groups, respectively. The implant surface was microtextured and smooth in 15.8% of the participants in the control group, and in 46.4%, 21.7%, and 37.5% of those in type 1, 2, and 3 groups, respectively. Regarding the ADM types, most of the participants in the control group used MegaDerm (63.9%), and most of those in type 1, 2, and 3 groups used CGCryoDerm (71.4%, 65.2%, and 79.2%, respectively). The incidence of capsular contracture was similar in all groups. There were two, one, and one infection case in the control, type 2, and type 3 groups, respectively. Thus, the clinical complications did not differ among the groups.

## 4. Discussion

This study evaluated the US findings of the ADM used in breast reconstruction and correlated them with histological findings. The ADM can present variably on US images. Most findings showed a constant thickness with homogeneous echotexture on the US images and homogeneous collagen fibers on the histology. However, focal thickening with decreased echogenicity, diffusely increased echogenicity, or bright echogenic spots within the ADM layer were also observed. Thus, we categorized three types of abnormal ADM findings and evaluated their correlations with histological findings. To our knowledge, there is only one case report describing the radiologic–pathologic correlation of ADM used in breast reconstruction [6].

US imaging is one of the most widely used imaging modalities in medicine because it is safe, portable, relatively inexpensive, and real-time examination can be performed. US imaging is most used for follow-up imaging in patients with breast reconstruction after mastectomy. The echo signal is generated from US wave interactions with tissue [22]. There are several interaction types, but reflection is the most important for tissue interactions and echo signal generation. An acoustic interface exists where two materials meet, and US reflections occur. The density difference between the two tissues affects the acoustic interface. Therefore, the greatest number of US waves are reflected between two tissues with a large density difference. Then, the US signal appears hyperechoic, which is bright white on a brightness mode (i.e., B-mode) display. Typically, most US waves are reflected at the interface between the soft tissue and air, or between the soft tissue and the bone or metal. Therefore, air, bone, and metal appear hyperechoic on ultrasonography.

**Figure 3:**
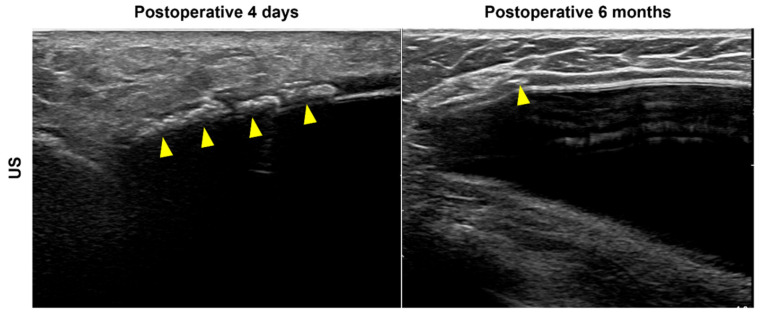
Acellular dermal matrix (ADM) ultrasound (US) findings in implant-based breast reconstruction. US imaging immediately after surgery (postoperative day 4) shows bright, hyperechoic dots diffusely visible in the ADM (arrowheads, left). After six months, the number of bright echogenic spots in the ADM reduced considerably (arrowhead, right).

In this study, we hypothesized a hyperechoic spot due to calcification inside the ADM for type 3 cases, but calcification was not histologically observed. Instead, multiple empty spaces were visible inside the ADM, perhaps because air was trapped in the empty spaces, appearing as bright hyperechoic dots in the US image. These empty spaces may be the result of skin appendage necrosis. Another possibility is the disappearance of fluid or fat during the process of creating the dermal matrix, where a cyst or fat necrosis in the donor tissue once was, entrapping air. For example, these bright, hyperechoic dots were diffusely observed in the ADM of a patient who received US imaging immediately after surgery (i.e., at 4 days postoperatively) (Figure 3). In the 6-month follow-up US examination, the number of bright echogenic spots in the ADM was considerably reduced (Figure 3).

**Figure 4:**
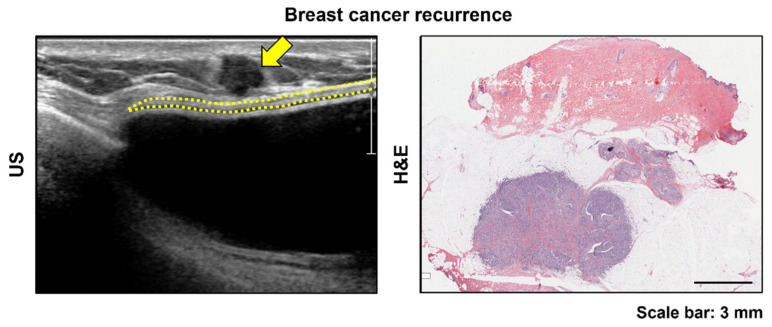
Cancer recurrence identified by ultrasound (US) examination after implant-based breast reconstruction. The image shows an irregular-shaped, hypoechoic mass (yellow arrow) located on the superficial aspect of the fibrous capsule (yellow dotted line). In this patient, the acellular dermal matrix layer was not visible on the US image. Histopathological examination indicated that the cancer mass was attached very close to the fibrous capsule.

Type 1 cases had focal thickening with decreased echogenicity on US imaging and patchy areas with chronic inflammatory infiltrate with a few foreign-body giant cells and neovascularization in the histological analysis. This type of US finding may be mistaken for cancer recurrence. Most of the malignant masses in the breast appear hypoechoic on US (Figure 4) due to tumor cells with surrounding stromal desmoplasia [23]. Most of the patients were referred to our hospital for a biopsy owing to suspected breast cancer recurrence after a US examination had type 1 findings. However, no patient had breast cancer recurrence in the ADM in our study.

Type 2 cases had diffusely increased echogenicity of the ADM on the US images and dense collagen bundles without degenerative or inflammatory changes and a few cells in the histology examination. If there is a dense collagen bundle, such as a tendon or ligament, it may appear hyperechoic on a US image [24].

The ADMs with normal US findings also showed slightly different histological appearances that seemingly reflect the donor’s condition, such as tissue degeneration or differences in the manufacturing process.

This study had several limitations. First, as this was a retrospective study, there might have been a selection bias. Second, breast US does not record whole-breast US images but analyzes only the images taken at the time of the examination. Thus, the analysis may be insufficient. Third, a biopsy was not performed for all patients with abnormal findings. However, there was no breast cancer recurrence in the ADM at 1-year follow-up examination in patients who did not undergo a biopsy.

## 5. Conclusions

We present the largest series of reports on ADM US findings and the only series with detailed imaging and pathological correlations. The ADM in implant-based breast reconstruction can have various US presentations. Most ADM cases present with a constant thickness with homogeneous echotexture on the US and homogeneous collagen fibers with neovascularization on the histology. However, focal thickening with decreased echogenicity, diffusely increased echogenicity, or bright echogenic spots within the ADM layer are possible. Some patients who underwent ADM biopsy for US abnormalities had benign results, such as focal inflammation, dense collagen, or empty spaces within the ADM, when histologically examined. Additionally, there was no case in which calcification was detected in the biopsy. As we analyzed US performed within a relatively early period after surgery (within 6 months to 1 year postoperatively), an additional study of long term follow-up US will be needed. This study is a reference for understanding ADM US image findings, which can help avoid unnecessary invasive procedures such as a biopsy or ADM removal.

## Figures and Tables

**Table 1:** Multiple logistic regression model of abnormal ultrasonographic findings in ADM risk factor.

	Control (%)	Type 1 (%)	Type 2 (%)	Type 3 (%)	OR	95% CI	*p* Value
No.	108	28	23	48			
Age at breast reconstruction, years							
Mean ± SD	45.5 ± 7.36	48.5 ± 9.14	51.6 ± 8.01	48.9 ± 7.74	1.074	1.022–1.129	0.005 *
BMI							
Mean ± SD	21.9 ± 2.87	23.1 ± 2.68	23.2 ± 3.06	23.5 ± 2.96	1.062	0.901–1.252	0.475

Chemotherapy	40 (37.0)	14 (50.0)	8 (34.8)	23 (47.9)	2.792	1.272–6.127	0.010 *
Radiotherapy	13 (12.0)	2 (7.1)	2 (8.7)	8 (16.7)	0.692	0.236–2.026	0.501

Implant volume, cc							
Mean ± SD	224.6 ± 83.58	255.9 ± 108.84	250.7 ± 113.25	260.4 ± 103.49	1.002	0.997–1.007	0.423
Implant surface							
Macrotextured	91 (84.3)	15 (53.6)	18 (78.3)	30 (62.5)	Reference		
Microtextured	3 (2.8)	7 (25.0)	3 (13.0)	10 (20.8)	5.403	1.284–22.736	0.021 *
Smooth	14 (13.0)	6 (21.4)	2 (8.7)	8 (16.7)	2.302	0.844–6.277	0.103

ADM type							
Megaderm	69 (63.9)	7 (25.0)	7 (30.4)	6 (12.5)	Reference		
CGCryoderm	29 (26.9)	20 (71.4)	15 (65.2)	38 (79.2)	8.167	3.722–17.922	<0.005 *
Alloderm	10 (9.3)	1 (3.6)	1 (4.3)	2 (4.2)	1.659	0.421–6.546	0.470
DermaCELL	0 (0.0)	0 (0.0)	0 (0.0)	2 (4.2)	N/A	0–0	1.000

Complication							
Capsular contracture	19 (17.6)	4 (14.3)	5 (21.7)	7 (14.6)	0.799	0.31–2.062	0.643
Infection	2 (1.9)	0 (0.0)	1 (4.3)	1 (2.1)	5.410	0.528–55.421	0.155
Necrosis	2 (1.9)	0 (0.0)	1 (4.3)	1 (2.1)	0.285	0.021–3.947	0.349
Wound dehiscence	6 (5.6)	1 (3.6)	0 (0.0)	2 (4.2)	0.542	0.069–4.233	0.559
Seroma	0 (0.0)	1 (3.6)	2 (8.7)	2 (4.2)	N/A	0–0	1.000

BMI, body mass index; ADM, acellular dermal matrix; SD, standard deviation; OR, odds ratio; CI, confidence interval; * *p* < 0.05.

## Data Availability

The data presented in this study are available on request from the corresponding author. The data are not publicly available due to consideration for patients’ privacy.
